# Childhood clumsiness and peer victimization: a case–control study of psychiatric patients

**DOI:** 10.1186/1471-244X-13-68

**Published:** 2013-02-25

**Authors:** Susanne Bejerot, Mats B Humble

**Affiliations:** 1 Department of Clinical Neuroscience, Karolinska Institutet, Stockholm, Sweden; 2 Psychiatric Research Centre, School of Health and Medical Sciences, Örebro University, Örebro, Sweden; 3 Northern Stockholm Psychiatry, St Göran Hospital, Stockholm, SE-112 81, Sweden

**Keywords:** Attention deficit disorder with hyperactivity, Autistic disorder, Bullying, Motor skills, Cerebellum, Movement

## Abstract

**Background:**

Poor motor and social skills as well as peer victimization are commonly reported in both ADHD and autism spectrum disorder. Positive relationships between poor motor and poor social skills, and between poor social skills and peer victimization, are well documented, but the relationship between poor motor skills and peer victimization has not been studied in psychiatric populations.

**Method:**

277 patients (133 males, 144 females), mean age 31 years, investigated for ADHD or autism spectrum disorder in adulthood and with normal intelligence, were interviewed about childhood peer victimization and examined for gross motor skills. The parents completed a comprehensive questionnaire on childhood problems, the Five to Fifteen. The Five to Fifteen is a validated questionnaire with 181 statements that covers various symptoms in childhood across eight different domains, one of them targeting motor skills. Regression models were used to evaluate the relationship between motor skills and the risk and duration of peer victimization, adjusted for sex and diagnosis.

**Results:**

Victims were described as more clumsy in childhood than their non-victimized counterparts. A significant independent association was found between reportedly poor childhood gross motor skills and peer victimization (adjusted odds ratio: 2.97 [95% confidence interval: 1.46-6.07], *n* = 235, *p* = 0.003). In adulthood, the victimized group performed worse on vertical jumps, a gross motor task, and were lonelier. Other factors that were expected to be associated with peer victimization were not found in this highly selected group.

**Conclusion:**

Poor gross motor skills constitute a strong and independent risk factor for peer victimization in childhood, regardless of sex, childhood psychiatric care and diagnosis.

## Background

Peer victimization [1] includes both confrontational behavior and relational forms of aggression (e.g., gossip and ostracism) and is a major problem in schools worldwide. At any given point, approximately 12% of children aged 8–13 are affected [2]. Associated risk factors for being victimized are low socioeconomic status, belonging to a minority group, being overweight, being perceived as deviant (e.g. physical appearance), learning disabilities, poor social skills (e.g., p rovocative behaviors and behaviors that indicate vulnerability) [3,4] and feeling lonely [1]. Victims are at risk for future psychiatric disorders including anxiety disorders, depression, suicidality, and psychosis [5-8]. However, it is uncertain whether peer victimization is the sole cause of these disorders or whether early signs of the disorder cause social skills deficits and subsequent victimization [9]. Undoubtedly, peer victimization increases the likelihood of becoming ill. A range of preventive methods has been used to reduce peer victimization, but most target the environment rather than focus on at-risk children [10].

Children with autism spectrum disorder (ASD) or attention-deficit hyperactivity disorder (ADHD) are at increased risk for being victimized by peers [11,12], presumably explained by the poor social skills that are integrated in the clinical presentation. However, poor social skills often coincide with poor motor skills in neurodevelopmental disorders [13], despite no direct linkage between global cognitive functioning and motor behavior [14]. Motor development status has gained little interest in psychiatric research but may provide a valuable link to understanding the underlying mechanisms or physiological components of mental states [15].

Children with emotional, behavioral, and pervasive developmental disorders often exhibit gross motor problems [16]. Approximately 50% of children with ADHD [17,18] and 60-80% of children with ASD have poor motor coordination [19,20] compared with 6% in the general population [21]. Conversely, poor motor skills have been shown to be a risk factor for anxiety [22,23]. In ADHD, coexisting clumsiness determines a particularly poor psychosocial prognosis [24] and is associated with more autistic traits [25]. Subtle motor signs in children tend to diminish before puberty. The persistence of motor signs into adulthood may indicate atypical neurological development [26].

In healthy subjects, an association between poor motor skills and peer victimization has been reported. However, these studies were based on retrospective reports from individuals at low risk for peer victimization and motor problems (i.e., healthcare workers across Sweden [27], Swedish university students [28], and Dutch youths [29]. Based on clinical experience, we hypothesized that poor motor skills and social skills deficits may represent different aspects of shared neurodevelopmental dysfunction [30-32]. In such a case, either or both could serve as risk factors for peer victimization and thereby constitute targets for preventive measures [28,29]. Possibly, assessments of motor skills problems are less stigmatizing and more reliable than those of poor social skills.

In the present study, psychiatric patients assessed for ASD or ADHD, disorders known to have a high prevalence of both motor and social skill problems in addition to peer victimization, were examined to explore the relationships between these factors. We hypothesized that there would be a positive association between peer victimization and poor motor skills, independent of psychiatric diagnosis and established risk factors for peer victimization.

## Method

### Participants

**Figure 1:**
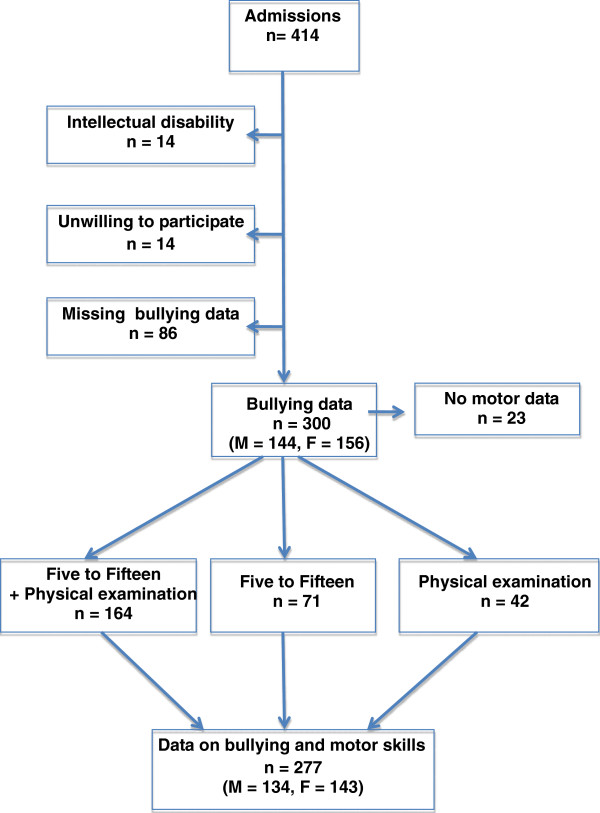
Flow chart of participants in the study.

All participants in this study were consecutive admissions referred to a specialized ASD and ADHD outpatient psychiatric clinic for diagnosing and treating adult ADHD or ASD at St. Göran Hospital, Stockholm, Sweden. Almost all of the patients were tertiary referrals from licensed psychiatrists or psychologists and almost half of the patients had received psychiatric care in childhood. No exclusion criteria were used but patients with obvious alcohol or drug dependence were referred elsewhere. Various treatment programs, including cognitive-behavioral therapy, support groups, medication, and courses about ADHD and ASD were provided to patients and their family members at the unit. A semi-structured protocol, including questions on civil status, educational level, employment status, current medication, psychiatric care in childhood and adulthood, and peer victimization, was utilized in the assessment of the patients. In 277 patients (133 males and 144 females, age range 18–57 years), information about peer victimization was available. In a subset of 235 individuals, a questionnaire on childhood symptoms was completed by a parent, and a motor function examination was performed in 206 patients (Figure 1).

### Neurodevelopmental assessment

All of the patients were assessed with a standardized research protocol to collect data on various aspects of adults with neurodevelopmental disorders. This included a diagnostic interview completed by a board-certified psychiatrist and psychologist. The assessment procedure took 12–18 h to complete over a period of 2–3 weeks. A parent, or in a few cases a significant other, was interviewed in person about early signs of ASD and ADHD based on the instrument Five to Fifteen (FTF) [33,34]. This questionnaire includes neuropsychiatric symptoms noted by parents when the child was between 5 and 15 years of age. It was sent home to the parent prior to the first visit and the results were discussed with the parent and patient together at the consultation with the psychiatrist. The FTF comprises 181 statements that can be endorsed as “does not apply” (= 0), “applies sometimes or to some extent” (= 1), or “definitely applies” (= 2). The items are organized into 22 subdomains that compose eight general domains: motor skills, executive functioning, perception, memory, language, learning, social skills, and emotional/behavioral problems. The “motor skills” domain contains two subdomains: gross motor skills and fine motor skills. Individual items are shown in the Appendix. In this study the parents’ endorsement of gross motor problems in childhood is equated with “clumsiness”, and social skills problems are equated with “poor social skills.”

### Assessment of peer victimization

Peer victimization was assessed using a specific question: “Have you ever been bullied?” If the answer was “Yes,” then the patient was asked whether the peer victimization was regarded as “a little” (i.e., notable to the patient but not necessarily known all over school) or “a lot” (i.e., clearly notable to people around) and during which time span it occurred (i.e., in nursery school, primary school [7–9 years old, 10–12 years old, 13–15 years old], or upper secondary school [16–19 years old]).

### Neuromotor examination

The psychiatrist conducted a physical examination that included motor function prior to determining the diagnosis of the patient. Gross motor coordination was observed during hopping with alternating vertical jumps and swinging the arms forward on the contralateral side. This was scored as “normal” (= 0; i.e., no difficulty accomplishing the task), “slightly abnormal” (= 1; i.e., difficulty coordinating the arms and legs), or “clearly abnormal” (= 2; i.e., great difficulty and only able to do at most three dis-coordinated hops). A score of 1 or 2 on this test was defined as “abnormal.”

### Diagnose specific assessments

A number of assessments were used for assisting in the diagnostic procedures for ASD and ADHD. The Autism Spectrum Screening Questionnaire (ASSQ), a 27-items screening tool for detecting high functioning children with ASD, was completed by a parent [35]. The Asperger Syndrome Diagnostic Interview (ASDI) [36], a highly structured diagnostic interview, comprising 20 different items including assessment of social impairment, narrow interests, repetitive routines, speech and language peculiarities, non-verbal communication problems, and motor clumsiness was used for almost all patients (*n* = 258). For a substantial group (*n* = 229) the self-rated Autism-Spectrum Quotient (AQ) [37] was also administered. All patients with a possible ADHD were assessed with a structured interview for ADHD, the Wender-Reimherr Adult Attention Deficit Disorder Scale (WRAADDS), which covers attention difficulties, hyperactivity/restlessness, temper, affective liability, emotional over-reactivity, disorganization, and impulsivity in adulthood [38]. The Wender Utah Rating Scale (WURS) was used in order to capture childhood symptoms of ADHD [39].

In addition, general functioning was assessed using the Global Assessment of Functioning (GAF) ranging from 0 to 100 [21], and depression was measured with the Montgomery-Åsberg Depression Rating Scale [40]. Wechsler Adult Intelligence Scale, neuropsychological version, (WAIS III and WAIS III-NI) was used in order to identify intellectual disability. In those few cases where WAIS was not administrated, a high educational level excluded mental retardation. Personality traits were assessed using the screening version of Structured clinical interview for DSM-IV (SCID II Screen) and with the Swedish universities Scales of Personality [41].

The diagnosis of ADHD or ASD was made after a consensus was reached between psychiatrist and psychologist according to the criteria of the *Diagnostic and Statistical Manual of Mental Disorders*, 4th Edition (DSM–IV criteria). Ninety-three patients (male, 49; female, 44) fulfilled the criteria for ASD, and 128 (male, 61; female, 67) fulfilled the criteria for ADHD. Fifty-six (male, 23; female, 33) had other severe psychiatric problems (e.g. chronic depression or severe anxiety disorders) (Table 1).

**Table 1:** Population characteristics in peer victimized and non-victimized patients

**Population characteristics**	**Victimized (*n*** **= 177)**	**Non- victimized (*n*** **= 100)**	***χ***^***2 ***^**( *d *. *f *.)**
Male, % (*n*)	50.9 (90)	43.0 (43)	
Female, % (*n*)	49.2 (87)	57.0 (57)	
Age, median (SD)	31.1 (10.5)	30.8 (10.3)	
Diagnosis			21.9 (2)***
Autism spectrum disorder, % (*n*)	43.5 (77)	16.0 (16)	
ADHD, % (*n*)	38.4 (68)	60.0 (60)	
Other psychiatric disorder, % (*n*)	18.1 (32)	24.0 (24)	
Poor gross motor skills in adulthood, % (*n*)^†^	38.4 (51)	21.9 (16)	5.8 (1)*
Poor gross motor skills in childhood, % (*n*)^††^	78.5 (117)	58.1 (50)	11.0 (1)***
Intelligence Quotient, total mean (SD) ^a^	98.8 (16.6)	98.1 (16.2)	
Body Mass Index, median (lower quartile; upper quartile) ^b^	23.7 (21; 27)	22.6 (20; 26)	
Civil status			
Single, % (*n*)	81.8 (139)	74.0 (74)	
Highest educational level, % (*n*) ^c^			
University	21.9 (37)	23.7 (23)	
Upper secondary school	32.0 (54)	21.7 (21)	
Vocational training	13.0 (22)	11.3 (11)	
Compulsory school	29.0 (49)	38.1 (37)	
Unfinished compulsory school	4.1 (7)	5.2 (5)	
Working full day (i.e., ≥ 70%), % (*n*) ^d^	16.7 (26)	17.4 (17)	
Seeing friends, % (*n*)			9.3 (2)**
> Weekly	36.9 (58)	50.0 (44)	
Once weekly to once monthly	29.3 (46)	34.1 (30)	
< Once monthly	33.8 (53)	15.9 (14)	
Previous childhood psychiatric care, % (*n*) ^e^	49.4 (81)	43.3 (42)	
Previous psychiatric hospitalization, % (*n*) ^f^	16.1 (26)	12.2 (12)	
Previous treatment in psychiatric care, median months (lower quartile; upper quartile) ^g^	24 (5; 72)	16 (1; 36)	
Previous depression, % (*n*) ^h^	46.7 (71)	36.3 (44)	
Present antidepressant treatment, % (*n*) ^i^	35.1 (46)	42.3 (33)	

^†^Assessed as vertical jump performance; ^††^retrospective report by parents using the Five to Fifteen questionnaire. Missing data: ^a^ = 82, ^b^ = 79, ^c^ = 11, ^d^ = 23, ^e^ = 16, ^f^ = 17, ^g^ = 27, ^h^ = 30, ^i^ = 68. SD, standard deviation; *n*, number. *p < 0.05, **p < 0.01, ***p < 0.001.

Data from this cohort on the outcome of central stimulant treatment in ADHD patients [42] and on psychiatric comorbidity in adults with ASD [43] have previously been reported. Approved written consent to use the information in research was obtained from the patients, and ethical approval was obtained from the Ethics Review Board, Karolinska hospital in Stockholm.

### Statistical analysis

The peer victimized group consisted of all participants who reported having been bullied in nursery school or school, regardless of severity and duration of the peer victimization. Dichotomous variables were analyzed using the Pearson *χ*^*2*^ test. Spearman’s rank correlation test was used to assess the correlations between motor skills and various domains on the FTF, with a significance level *p* < 0.001.

A bivariate logistic regression analysis, with peer victimization as the dependent variable, was used to screen the subdomains of the FTF (Table 2). The analyses that showed significant relationships with peer victimization were included in a multivariate logistic regression analysis, in which risk factors indicated from previous research (i.e., sex, diagnosis, and psychiatric care in childhood) were also included. For all of these analyses, the mean FTF subdomain scores (ranging from 0 to 2) were used. In the logistic regressions, odds ratios were calculated as unit change (i.e., the changed likelihood that resulted from an increase of subdomain score by 1). In a third step, the number of time spans of peer victimization, gross motor skills problems according to the FTF, sex, ASD, ADHD, and other diagnosis, and sex × diagnoses interactions were analyzed in an ordinal regression model (polytomous universal model). The significance level was set to *p* < 0.05. Statistica version 10 software (StatSoft) was used for all of the analyses, with the exception that SPSS version 19 software was used for the ordinal regression (PLUM).

**Table 2:** **Five to Fifteen scores retrospectively reported by the parents of 235 adult psychiatric patients and odds ratios for peer victimization, bivariately unadjusted and adjusted (*n*** **= 210) for sex, diagnosis (ASD, ADHD, or other), previous child psychiatric treatment, and educational level**

	**Victimized**	**Non- victimized**	**Odds Ratio**	***p***	**Odds Ratio**	***p***
	***n*** **= 149**	***n*** **= 86**	**Unadjusted,unit change**		**Adjusted,unit change**	
	**(male, 79; female, 70)**	**(male, 38; female, 48)**	**(95% confidence intervals)**		**(95% confidence intervals)**	
	***Group means *(± *SD*) *of subdomain mean scores***					
**Five to Fifteen subdomain**						
Gross motor skills	0.60 (0.57)	0.40 (0.50)	1.97 (1.16-3.35)	0.009	2.97 (1.46-6.07)	0.003
Fine motor skills	0.39 (0.46)	0.31 (0.40)	1.55 (0.83-2.92)	0.16		
Attention	0.89 (0.65)	1.02 (0.70)	0.74 (0.50-1.11)	0.15	0.43 (0.20-0.93)	0.033
Hyperactive/Impulsive	0.46 (0.52)	0.61 (0.64)	0.64 (0.33-1.24)	0.18		
Hypoactive	1.07 (0.61)	0.94 (0.60)	1.44 (0.77-2.70)	0.25		
Planning and organizing	0.88 (0.71)	0.90 (0.78)	0.97 (0.67-1.39)	0.86		
Relation in space	0.34 (0.46)	0.26 (0.39)	1.59 (0.82-3.09)	0.16		
Time concepts	0.39 (0.51)	0.42 (0.52)	0.91 (0.54-1.54)	0.72		
Body perception	0.45 (0.52)	0.40 (0.46)	1.25 (0.72-2.17)	0.42		
Visual perception	0.25 (0.39)	0.15 (0.33)	2.22 (0.95-5.19)	0.049	2.06 (0.75-5.66)	0.16
Memory	0.50 (0.45)	0.43 (0.42)	1.43 (0.76-2.69)	0.26		
Language comprehension	0.42 (0.56)	0.47 (0.59)	0.93 (0.58-1.49)	0.76		
Expressive language skills	0.22 (0.31)	0.15 (0.23)	2.45 (0.86-7.02)	0.079	2.12 (0.51-8.84)	0.30
Language communication	0.52 (0.63)	0.47 (0.63)	1.16 (0.75-1.79)	0.50		
Reading/Writing	0.44 (0.48)	0.49 (0.53)	0.84 (0.39-1.79)	0.64		
Math	0.56 (0.67)	0.65 (0.76)	0.84 (0.49-1.44)	0.52		
General learning	0.80 (0.67)	0.72 (0.72)	1.18 (0.79-1.76)	0.41		
Coping in learning	0.83 (0.58)	0.86 (0.59)	0.91 (0.58-1.45)	0.70		
Social skills	0.58 (0.44)	0.48 (0.44)	1.68 (0.89-3.14)	0.10		
Internalizing	0.66 (0.49)	0.71 (0.54)	0.82 (0.39-1.73)	0.60		
Externalizing	0.34 (0.37)	0.57 (0.49)	0.30 (0.12-0.74)	0.007	0.51 (0.19-1.36)	0.18
Obsessive-compulsive	0.27 (0.33)	0.27 (0.37)	1.04 (0.47-2.26)	0.93		
Male sex			1.37 (0.84-2.25)	0.21	1.44 (0.74-2.79)	0.28
ASD			4.04 (2.19-7.47)	0.00001	3.14 (1.47-6.73)	0.003
ADHD			0.42 (0.25-0.69)	0.0007	1.47 (0.67-3.22)	0.33
Previous child psychiatric treatment			1.28 (0.77-2.12)	0.34	1.52 (0.78-2.96)	0.22
Educational level			1.57 (0.71-3.49)	0.27	0.76 (0.24-2.35)	0.63

The Five to Fifteen subdomain scores range between 0–2. Higher scores indicate more problems. Odds ratios represent the changed likelihood of peer victimization, resulting from a 1 score step increase on the mean of the Five to Fifteen subdomain.

## Results

### Peer victimization rates

Of the total sample, 64% (68% of the males and 60% of the females) reported that peers had victimized them in childhood. Most afflicted were individuals diagnosed with ASD, 83% of whom had been victimized. This rate was markedly higher than the rate among those with ADHD (53%) or those with other psychiatric diagnoses (57%). Of those who were victimized at any time period, 69% were also victimized during at least one additional period, confirming the repetitiveness of the experience.

In our sample, education, civil status, employment status, GAF, Body Mass Index in adulthood, Intelligence Quotient, current depressive symptoms, antidepressant treatment, and history of childhood psychiatric care did not differ between the victims and non-victims groups. However, those with a history of being victimized were lonelier compared with the non-victimized group (Table 1).

### Risk for peer victimization in relation to childhood neuropsychiatric symptoms

**Figure 2:**
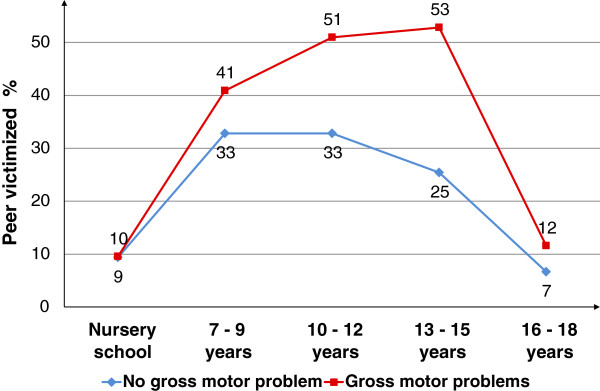
**Peer victimization among 235 psychiatric patients during consecutive time periods in childhood, separated by gross motor skills according to retrospective reports by parents.** *χ^*2*^ (*df* = 1) = 6.04,, *p* = 0.014; ***χ^*2*^ (*df* = 1) = 13.7, *p* = 0.0002.

In 235 participants, information on childhood symptoms was retrospectively obtained from a parent. Sixty-eight patients were reported to not have had any gross motor skill problems in childhood, whereas in 167 patients clumsiness was reported. Seventy per cent of the individuals with reportedly poor gross motor skills had been victimized by peers compared with 47% of those with normal motor skills in childhood, corresponding to an unadjusted odds ratio of 2.63 (95% confidence interval: 1.47-4.70). Girls who were reported to have poor motor skills had the highest risk for being victimized by peers at 10–12 years of age compared with a peak risk at 13–15 years of age for boys with poor motor skills (data not shown). Peer victimization at the different age spans is presented in Figure 2 for those with and without reported gross motor skills problems in childhood.

Impaired visual perception was similarly associated with an increased risk for peer victimization (unadjusted odds ratio 2.00, 95% confidence interval: 1.10-3.63); however, none of the other domains of the FTF was associated with an increased odds ratio for peer victimization in the bivariate regression. In this particular cohort, externalizing and attention problems reported on the FTF, suggesting ADHD, seemingly decreased the risk of being victimized. In Table 2, the odds ratios for peer victimization associated with the various FTF domains (as continuous variables), sex, previous child psychiatric treatment, diagnosis, and educational level are presented, showing an almost three-fold increased risk of being victimized by peers among those with poor gross motor skills in childhood.

In an ordinal logistic regression model, with the number of time spans of peer victimization as the dependent variable, poor gross motor skills in childhood remained an independent risk factor when sex, diagnosis (i.e., ADHD, ASD, and other), and sex × diagnosis interactions (which turned out non-significant) were introduced in the model. The association between poor gross motor skills in childhood and number of peer victimization periods had an odds ratio of 1.78 (95% confidence interval: 1.13-2.79; *n* = 227, *p* = 0.013).

### Correlations between motor problems, visual perception and other childhood symptoms, as measured with the Five to Fifteen

Strong correlations were found between gross motor skills in childhood and body perception (*ρ* (*n* = 232) = 0.57, *p* < 0.0001), fine motor skills (*ρ* (*n* = 235) = 0.55, *p* < 0.0001), and social skills in childhood (*ρ* (*n* = 233) = 0.51, *p* < 0.0001). Visual perception correlated strongly with fine motor skills (*ρ* (*n* = 230) = 0.55, *p* < 0.0001) and body perception (*ρ* (*n* = 228) = 0.53, *p* < 0.0001). Weaker correlations, but highly significant, were found between vertical jumps in adulthood and gross motor skills in childhood (*ρ* (*n* =168) = 0.26, *p* = 0.0008), body perception (*ρ* (*n* =166) = 0.29, *p* = 0.0002), and social skills in childhood (*ρ* (*n* =168) = 0.25, *p* = 0.0009).

### Gross motor dysfunction in relation to peer victimization and diagnosis

Of those with impaired gross motor function (i.e., poor vertical jump performance), 75% had been victimized by peers in childhood compared with 59% of those with normal performance (*χ*^*2*^ (*df* =1) = 6.01, *p* = 0.01, *n* = 206, unadjusted odds ratio 2.22 [95% confidence interval: 1.15-4.28]).

**Figure 3:**
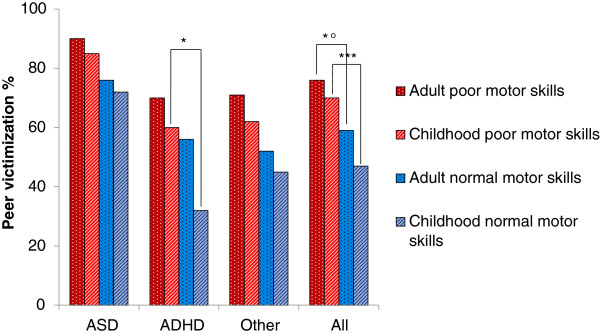
**Association between diagnostic group, gross motor skills and per cent peer victimization.** *χ^*2*^ (*df* = 1) = 5.80, *p* = 0.016; *°*χ*^*2*^ (*df* = 1) = 6.46, *p* = 0.011; ***χ^*2*^ (*df* = 1) = 11.0, *p* = 0.0009.

A larger proportion of patients with ASD performed poorly on vertical jumps compared with those with ADHD or other diagnoses (*χ*^*2*^ (*df* =2) = 8.11, *p* < 0.05, *n* = 205), showing impairments in 44.0% of the ASD subjects, 29.0% of the ADHD subjects, and 18% of the subjects with other diagnoses. However, the relationship between peer victimization and poor motor skills was not dependent on the ASD diagnosis (Figure 3).

## Discussion

In this study, we showed that childhood motor clumsiness, reported by parents, and poor performance in a simple motor test, assessed in adulthood, are strongly associated with childhood peer victimization, as reported by adult psychiatric patients. This association remained when diagnosis was controlled for. The present study extends previous findings to psychiatric patients at substantial risk for social and motor skills problems and peer victimization. Additionally, the clumsiness retrospectively reported by the parents was often observable in adulthood as poor vertical jump performance, suggesting deviant cerebellar development. Moreover, impaired visual perception, but none of the other domains of the FTF, was similarly associated with increased odds for peer victimization. A strong link between motor and visuospatial deficits exists both in the general population and in clumsy children [44]. Thus the fact that "gross motor skills" and "visual perception" were the only two domains from the FTF that showed an association with increased peer victimization strengthens our findings.

### Cerebellar dysfunction in neurodevelopmental disorders

Motor deficits in children with ADHD and ASD are well documented [30,45,46], and comorbidity between ASD, ADHD, and developmental coordination disorder has long been recognized [13]. Children with a combination of motor coordination deficits and ADHD have less favorable outcomes and more autistic traits [24], including perceptual problems, supporting the concepts of Deficits in Attention, Motor control, and Perception (“DAMP”) [25,30] and Early Symptomatic Syndromes Eliciting Neurodevelopmental Clinical Examinations (“ESSENCE”) [47]. This also supports the idea that atypical brain development can explain the interrelatedness between these disorders [48].

Although motor abnormalities are common in ADHD and ASD and pivotal for the diagnosis of developmental coordination disorder, the underlying neurophysiologic impairments and type and persistence of motor problems appear to vary between the diagnostic groups and subgroups. Research in adults with ASD suggests a combination of cerebellar and basal ganglia deficits [49], and imaging studies of ASD and ADHD have consistently found cerebellar abnormalities [50-52]. The cerebellum controls sensorimotor coordination and is critical to timing computations in both motor and non-motor tasks [53]. Working memory, the shifting of attention, implicit learning, emotional regulation, executive function and facial recognition all depend on cerebellar functioning [50,54]. Moreover, symptoms of cerebellar lesions resemble the typical impairments of ASD [55].

In this study, the parents reported poor social skills in the majority of the cases (victims and non-victims), consistent with the social deficits related to ASD and to some extent to ADHD. However, poor gross motor skills in childhood but not poor social skills predicted peer victimization in the present study. Because poor social skills are frequent in both ADHD and ASD [56,57], this by itself may explain the increased risk for peer victimization compared with normal controls [11,12,58]. Nevertheless, we propose that poor gross motor functioning constitutes an independent risk factor for peer victimization across various populations, regardless of provocative behaviors or anxiousness. Poor motor skills may manifest as impaired social skills, thereby contributing to peer victimization. Success in social interaction is largely a matter of timing and harmonious integration of verbal and non-verbal cues. In other words, cerebellar function may be critical for social success. Impairments in social functioning could be viewed as disintegrated motor skills, reflected by subtle aberrations in facial expressions, gaze, pitch, prosody, wording, posture, gestures, turn-taking, and sense of appropriate physical distance. Even slight signs of impairment, registered at a subliminal level, may affect others’ judgments and determine whether the person will be viewed as socially “normal” and attractive as a companion or considered “strange” and result in social exclusion or peer victimization. Preadolescents and adolescents are exceptionally sensitive to deviations [59], are easily embarrassed [60], and strive to conform to their peers [59,61], possibly explaining why peer victimization peaks in the preteen years [62]. We suggest that motor skills are associated with social likeability and that poor skills may contribute to the loneliness in adulthood reported by the victims in this study. Thus, the feeling of loneliness often reported by bullied children [1] seems to continue into adulthood.

Another possible explanation for our findings could be the close association between poor motor skills and visual perceptual dysfunction. Visual perceptual dysfunction has been shown to be associated with the number of co-occurring disorders in children with developmental coordination disorder [63] and contribute to poor outcome in preterm born babies [64] and in children with hyperopia [65]. Poor motor skills without visual perception dysfunction may represent a dimension of normality whereas the combination of the two reflects a neurological dysfunction with increased risk for social exclusion, and/or cognitive, behavioral and emotional disability. In a study on adults with either social phobia or obsessive-compulsive disorder (OCD), childhood peer victimization was much more often reported in the OCD group [66]. Although both disorders have similar ages of onset, chronicity, and relationships with avoidant personality, OCD differs from social phobia by being associated with motor problems and soft neurological signs [67-69], including deficits in visuospatial skills [70]. Interestingly, poor visuospatial skills predicted persistence in pediatric-onset OCD [71].

### Associated risk factors

None of the established factors previously shown to be associated with peer victimization (e.g., education level, civil status, employment, general functioning, overweight, intelligence, reported social skills, internalization, depression, and antidepressant treatment) were implicated in this study. Notably, however, childhood Body Mass Index data were not obtained. By studying a sample at high risk for peer victimization, risk factors beyond the expected may be revealed, such as poor gross motor skills. Hence, our lack of findings about the established risk factors cannot be generalized to normally developing children.

### Clinical implications

Psychiatric patients are often targets of peer victimization in childhood. Bullying amongst normally developing children peaks in the pre teens, but children with both poor gross motor skills and psychiatric problems have often been bullied for a prolonged period of time. Prolonged peer rejection tends to result in poor self-esteem and may decrease the ability to appeal to others. In adult psychiatric patients a history of poor gross motor skills and peer victimization in childhood may indicate a severe and pervasive neuropsychiatric disorder with social skills deficits. In such cases the clinician should be aware that a range of support and treatments often are indicated.

Simple assessments of motor skills in pre school children should be helpful for identifying children at risk. Furthermore, the possibility should be explored whether early intervention programs using specialized physical education adapted for ‘clumsy’ children (e.g. [72]) reduce the risk of loneliness and peer rejection.

### Study limitations

The patient group studied here may be perceived as marginal; on the other hand ADHD is not by any means a rare disorder in adults. ADHD has a chronic course in approximately 4% of the adult population [73] and can be diagnosed in almost one of four adult psychiatric outpatients [74,75]. Concerning ASD, the prevalence rate in the general population exceeds 1% [76], and ASD frequently co-occurs with other psychiatric disorders. In a recent study, 70% of adults with ASD had experienced at least one episode of major depression [77]. Possibly, the co-occurring disorders draw the clinician’s attention, whereas ADHD and ASD often remain un-diagnosed.

Nevertheless, this study has several limitations, partially due to lack of relevant instruments and the naturalistic setting. First, our retrospective use of FTF could interfere with the results, as the FTF was designed to be filled out by parents, when evaluating their children. Consequently, since parents may forget earlier signs of problematic behavior, false-negative responses may constitute a problem. However, false positive results seem less likely. In our experience, the parents recalled signs of atypical development remarkably well and our results are consistent with a previous report on children with ASD and ADHD that used the FTF [18]. Secondly, we did not use any specific definition of peer victimization, but the concept of bullying is well known [78], and the patients never questioned the meaning of the wording. They knew whether or not they were victimized by peers and remembered how long it lasted and who where the perpetrators. Being victimized by peers is such a traumatic experience that it may reside as a humiliating memory throughout one’s life. Although some children may deny being victimized by peers or belittle it as “teasing”, we question whether this bias prevails into adulthood. In fact, self-reports of peer victimization by adults may be more reliable than children’s self-reports because the shame that hindered them in childhood may eventually subside. In this study we did not inquire about type of bullying that the participants had experienced, which is another limitation. Most of the participants were in their preteens during the eighties and nineties, thus cyber bullying had not emerged on the scene yet. The three-point scale to determine severity used in this study is not a validated method for measuring severity. However, the question is straightforward and the responses were dichotomized in the analyses; thus the peer victim group consisted of all participants who reported being bullied, regardless of severity. Attrition was partially attributed to the fact that many patients were originally investigated at other clinics and only referred to us for medication, thus providing a smaller proportion of patients to examine. Of the total sample, 21% were not included in the study because of missing data. However, no significant demographic differences were found between these individuals and those included in the study.

## Conclusion

Some children are persistently victimized during childhood, which is associated with poor mental health. Risk factors for peer victimization need to be identified to protect the most vulnerable children from feeling rejected, offended, and increasingly marginalized. In this study, poor gross motor skills in childhood predicted peer victimization among psychiatric patients assessed for ADHD or ASD in adulthood. We propose that poor motor functioning constitutes an independent and strong risk factor for peer victimization. Conceivably, subclinical cerebellar dysfunctions are perceived by others at a subliminal level. Subtle dys-coordinated behaviors may then give the impression of “awkwardness” and not fitting in, leading to peer rejection.

These findings may be useful for developing methods to protect and empower vulnerable children, which is a challenge for future research emphasizing the need for longitudinal intervention studies.

## Appendix

### 

Five to Fifteen (FTF)

### 

Gross motor skills

### 

Difficulty acquiring new motor skills

Difficulty throwing and catching a ball

Difficulty running fast and smoothly

Difficulty/does not like to participate in game sports

Has balance problems

Often trips and falls

Has clumsy movements

### 

Fine motor skills

### 

Has difficulty drawing

Difficulty manipulating small objects

Difficulty pouring water into a glass

Often spills food onto clothes when eating

Difficulty using knife and fork

Difficulty buttoning buttons/tying shoe-laces

Difficulty using a pen

Has not developed clear hand-dominance

Writing is slow and awkward

Has immature pen-grip

## Competing interests

The authors have no competing interests to declare in relation to the manuscript.

## Authors’ contributions

SB designed the study. SB and MH together drafted the manuscript. Both authors approved the final manuscript.

## Pre-publication history

The pre-publication history for this paper can be accessed here:

http://www.biomedcentral.com/1471-244X/13/68/prepub
